# Transcatheter aortic valve replacement during the COVID‐19 pandemic—A Dutch single‐center analysis

**DOI:** 10.1111/jocs.15123

**Published:** 2020-10-21

**Authors:** Maxim J.P. Rooijakkers, Wilson W.L. Li, Laurens W.L.M. Wollersheim, Guillaume S.C. Geuzebroek, Helmut Gehlmann, Leen A.F.M. van Garsse, Marleen H. van Wely, Michel W.A. Verkroost, Wim J. Morshuis, Heiman Wertheim, Niels van Royen

**Affiliations:** ^1^ Department of Cardiology Radboud University Medical Center Nijmegen The Netherlands; ^2^ Department of Cardiothoracic Surgery Radboud University Medical Center Nijmegen The Netherlands; ^3^ Department of Medical Microbiology Radboud University Medical Center Nijmegen The Netherlands

**Keywords:** replacement, valve repair

## Abstract

**Background and Aim of the Study:**

The coronavirus disease 2019 (COVID‐19) pandemic has put an enormous strain on healthcare systems and intensive care unit (ICU) capacity, leading to suspension of most elective procedures, including transcatheter aortic valve replacement (TAVR). However, deferment of TAVR is associated with significant wait‐time mortality in patients with severe aortic valve stenosis. Conversely, there is currently no data available regarding the safety and feasibility of a continued TAVR program during this unprecedented crisis. The aim of this study is to evaluate the safety and feasibility of patients undergoing TAVR during the COVID‐19 pandemic in our center, with specific emphasis on COVID‐19 related outcomes.

**Methods:**

All patients who underwent TAVR in our center between February 27, 2020, and June 30, 2020, were evaluated. Clinical outcomes were described in terms of Valve Academic Research Consortium 2 definitions. Patient follow‐up was done by chart review and telephone survey.

**Results:**

A total of 71 patients have undergone TAVR during the study period. Median age was 80 years, 63% were men, and 25% were inpatients. Procedural success was 99%. After TAVR, 30% involved admission to the ICU, and 94% were ultimately discharged to the cardiac care unit on the same day. Two patients (3%) had confirmed COVID‐19 a few days after TAVR, and both died of COVID‐19 pneumonia within 2 weeks after hospital discharge.

**Conclusions:**

A continued TAVR program during the COVID‐19 pandemic is feasible despite limited hospital resources. However, COVID‐19 related mortality after TAVR is of concern.

## 1 INTRODUCTION

The coronavirus disease 2019 (COVID‐19) pandemic^1^ has put an enormous strain on existing healthcare systems and resources worldwide, leading to the deferment of most elective procedures.^2^, ^3^ This especially affects patients with severe aortic valve stenosis (AS) awaiting transcatheter aortic valve replacement (TAVR), a recognized vulnerable population with established cardiovascular disease and important comorbidities. There is general consensus that the deferment of these potentially life‐saving procedures is associated with dangers of sudden cardiac death or irreversible cardiac deterioration. For example, it has been reported that there are important wait‐time mortality risks of 23.3% and 27.5%, respectively at 6‐ and 12‐month awaiting TAVR.^4^

The American College of Cardiology (ACC) and Society of Cardiac Angiography and Interventions (SCAI) have recently published a consensus statement regarding triage considerations for patients referred for structural heart disease intervention during the COVID‐19 crisis, including when to perform TAVR for severe symptomatic AS.^5^ However, the risks of adverse events caused by postponement of these interventions should be balanced against the additional COVID‐19 related dangers of performing (high‐risk) cardiovascular interventions during this global crisis. Unfortunately, there is currently a paucity of data to properly estimate the additional hazards of TAVR during the COVID‐19 pandemic, especially regarding the risks of COVID‐19 transmission just before or after the intervention (either through healthcare workers, visitors, or other patients), but also regarding COVID‐19 related morbidity and mortality. Therefore, we performed the current study to evaluate the feasibility and safety of a continued TAVR program during the COVID‐19 pandemic in the Netherlands, evaluating early clinical results with specific emphasis on COVID‐19 related outcomes.

## 2 MATERIALS AND METHODS

We conducted a single‐institutional cohort study evaluating all patients who underwent TAVR in our center for various urgency indications (Table 1) during the COVID‐19 period in The Netherlands. The local Medical Research Ethics Committee provided a waiver since the study does not require an ethical review. Informed consent was also waived by the aforementioned Committee. The study was conducted in accordance with the principles of ICH Good Clinical Practice, applicable privacy requirements, and guiding principles of the Declaration of Helsinki. The study period began on February 27, 2020, with the identification of the first COVID‐19 patient in the Netherlands, and ended June 30, 2020. Up until September 17, 2020, more than 84,000 patients have been tested positive for COVID‐19 in the Netherlands with a total population of 17 million inhabitants, necessitating more than 12,300 hospital admissions and leading to more than 6200 deaths.^6^ In our hospital, we have admitted more than 330 patients with COVID‐19. At the peak of the pandemic, we had 48 patients admitted on the COVID‐ 19 ward and 41 patients on the intensive care unit (ICU) on a single day. There is a healthcare worker COVID‐19 screening program where personnel can be tested on a daily basis in case of suspect symptoms.^7^ Just over 240 hospital employees have tested positive at our institution and most have returned to work. Most common risk factor for COVID‐19 was outside the hospital setting (ski trip, carnival festivities, or a household member positive).

**Table 1:** Urgency categories for patients with severe aortic valve stenosis awaiting transcatheter aortic valve replacement

Timing intervention	<1 Week	<1 Month	<3 Months
Indication	Life‐threatening in case of deferral of procedure	Potentially life‐threatening or negatively impacting prognosis in case of deferral of procedure for more than 1 month	Limited impact on prognosis in case of deferral of procedure for more than 1 month
Symptomatology	Severely symptomatic	Moderately symptomatic	Mildly symptomatic
Complaints	Heart failure requiring hospitalization, severe orthopnea	Syncope due to AS, dizziness	NYHA I–II, anginal complaints
Echocardiography	Critical AS (AVA <0.6 cm^2^ and/or mean gradient >60 mmHg) or severe AS (AVA <1.0 cm^2^ and/or mean gradient >40 mmHg)	Critical AS (AVA <0.6 cm^2^ and/or mean gradient >60 mmHg) or severe AS (AVA <1.0 cm^2^ and/or mean gradient >40 mmHg)	Severe AS (AVA <1.0 cm^2^ and/or mean gradient >40 mmHg)

Abbreviations: AS, aortic valve stenosis; AVA, aortic valve area.

### 2.1 Patient triage

With progression of the COVID‐19 crisis and the increasing COVID‐19 caseload on the ICU, our center started with deferral of all nonurgent procedures on March 16, 2020. From that time, all patients on the TAVR waiting list were triaged on a daily basis by a single TAVR cardiologist (MvW), based on an urgency classification system categorized into three levels (Table 1): Levels 1, 2, and 3, indicating TAVR to be performed preferably within 1 week, 1 month, or 3 months, respectively. Level of urgency was mainly determined by symptom severity and echocardiographic features (Table 1). Our classification is largely comparable with the later published ACC/SCAI consensus statement.^5^ The consenting process to undergo a TAVR procedure during the COVID‐19 pandemic did not differ significantly from the one in the pre‐COVID‐19 era. However, the risk of a possible COVID‐19 infection was well weighed against the risk of postponing the TAVR procedure. In the pre‐COVID‐19 era our hospital performed on average five TAVR procedures per week, divided over two working days. From the beginning of this pandemic, along with the progressive restriction on hospital resources, our hospital strived to perform one TAVR procedure on each regular working day. By spreading the TAVR procedures, the burden on anesthesia personnel, coronary care unit and ICUs was reduced.

### 2.2 Periprocedural COVID‐19 screening and management

All patients were screened for COVID‐19 symptoms and contacts with COVID‐19 suspected individuals. For outpatients, this was performed by telephone survey. From April 6, 2020, routine preoperative COVID‐19 screening with polymerase chain reaction (PCR) testing was commenced in our institution for high complex surgical procedures. For patients planned for TAVR under general anesthesia, as well as procedures planned under conscious sedation but with a high risk for conversion to general anesthesia, PCR testing was performed less than 48 h before procedure. In patients planned for TAVR under conscious sedation without high risk for conversion and without suspicion of COVID‐19, no PCR testing was performed. TAVR was deferred for all patients with possible COVID‐19 symptoms and/or a positive PCR.

TAVR was performed according to routine protocol. Procedures were performed in a hybrid catheterization laboratory with a standard operating team consisting of an interventional cardiologist, cardiothoracic surgeon, imaging cardiologist, and anesthesiologist. When endotracheal intubation was performed, only the anesthesiologist and necessary (anesthesia) personnel were present in the room, dressed in isolation gowns, FFP‐2 masks and face shields. During the TAVR procedure, routine universal precautions and personal protective equipment usage were followed, including standard surgical masks. After the procedure, patients were transferred to the cardiac care unit (CCU), unless admission to the ICU was indicated. During the study period, patients were transferred to a separate, newly created non‐COVID‐19 ICU when indicated. During the postprocedural period in the ICU, routine protective measures were followed, including plastic nonreusable gowns and nonsterile gloves for all personnel. There was no personnel interchange during work shifts between the non‐COVID‐19 ICU and the specific COVID‐19 ICUs.

Patients were routinely transferred 1 day after TAVR to their referring hospital for further rehabilitation. Both the TAVR protocol and our study protocol did not change over time. Since the TAVR population, in general, is an elderly, vulnerable population, most patients who underwent a TAVR procedure presumably stayed mostly at home during the first postoperative period to prevent a possible COVID‐19 infection. However, we have no data available on this.

### 2.3 Data collection and follow‐up

Data collection was performed by chart review from our institution as well as the referring center. In addition, all patients underwent telephone follow‐up at least 2 weeks after definitive hospital discharge. Clinical endpoints were prospectively collected according to the updated Valve Academic Research Consortium 2 (VARC‐2) criteria.^8^

### 2.4 Statistical analysis

Categorical variables are presented as numbers with percentages and frequencies. Continuous variables are presented as median with interquartile range (IQR). Descriptive statistics were performed using IBM SPSS Statistics software version 25.0 (IBM Corp).

## 3 RESULTS

During the study period, 71 consecutive TAVR procedures were performed (Table 2). Median age in this cohort was 80 years (74–84) and 63% were men. All patients were referred for TAVR due to symptomatic, severe AS, except for one case who was referred because of severe aortic regurgitation. Before TAVR, patients had median New York Heart Association (NYHA) functional Class II, with 28 patients (39%) being in NYHA Class III–IV. Median left ventricular ejection fraction (LVEF) was 55%. A total of 18 patients (25%) were inpatients, admitted with heart failure or syncope due to severe AS. The majority of patients were outpatients who were highly symptomatic with either severe or critical AS (Table 2).

**Table 2:** Patients' baseline characteristics

Demographics	Study population N=71
Age, years	80 (74–84)
Male gender, *n* (%)	45 (63)
Body mass index (BMI), kg/m^2^	27 (24–30)
Obesity (BMI >30), *n* (%)	17 (24)
Current smoker, *n* (%)	4 (6)
Medical history	
Hypertension, *n* (%)	49 (69)
Diabetes mellitus, *n* (%)	26 (37)
Coronary artery disease, *n* (%)	49 (69)
Previous myocardial infarction, *n* (%)	16 (23)
Previous percutaneous coronary intervention, *n* (%)	32 (45)
Previous cardiac surgery, *n* (%)	16 (23)
Peripheral artery disease, *n* (%)	15 (21)
Previous stroke/TIA, *n* (%)	14 (20)
Creatinine >2 mg/dl, *n* (%)	5 (7)
MDRD‐GFR, ml/min	63 (50–76)
Liver disease, *n* (%)	1 (1)
Current/previous malignancy, *n* (%)	16 (23)
COPD, *n* (%)	15 (21)
Atrial fibrillation, *n* (%)	27 (38)
Prior pacemaker/ICD implantation, *n* (%)	1 (1)
EuroSCORE II	2.4 (1.5–4.4)
EuroSCORE, logistic	11.7 (7.0–22.9)
NYHA class	2 (2–3)
NYHA Class III/IV, *n* (%)	28 (39)
Preoperative screening by geriatrist, *n* (%)	49 (69)
Frailty	
Not frail, *n* (%)	49 (69)
Mildly frail, *n* (%)	18 (25)
Moderately frail, *n* (%)	4 (6)
Urgency level of TAVR procedure	
Urgency 1 (TAVR <1 week), *n* (%)	17 (24)
Urgency 2 (TAVR <1 month), *n* (%)	30 (42)
Urgency 3 (TAVR <3 months), *n* (%)	24 (34)
Inpatient/outpatients, *n* (%)	18/53 (25/75)
Echocardiographic variables	
Left ventricular ejection fraction, %	55 (45–60)
Aortic valve area, cm^2^	0.8 (0.6–0.9)
Mean gradient, mmHg	40 (30–49)
Moderate or severe mitral regurgitation, *n* (%)	9 (13)

*Note*: Data are presented as median with interquartile range, or as number (%).

Abbreviations: COPD, chronic obstructive pulmonary disease; ICD, implantable cardioverter defibrillator; MDRD‐GFR, modification of diet in renal disease—glomerular filtration rate; NYHA, New York Heart Association; TAVR,  transcatheter aortic valve replacement; TIA, transient ischemic attack.

Conscious sedation was planned in 43 patients (61%), of which one patient required conversion to general anesthesia due to failure of the vascular closure device, necessitating surgical repair of the femoral artery. Other VARC‐2‐defined outcomes (Table 3) included 6 patients (8%) requiring a permanent pacemaker implantation due to conduction disturbances, 8 patients (11%) with either a minor or major vascular complication, 8 patients (11%) with either a minor or major bleeding and 1 patient (1%) with conversion to open surgery due to luxation of the device in the ascending aorta. There was one procedural death: the patient had severe left ventricular dysfunction at the start of the procedure, which was significantly deteriorated when compared to a transthoracic echocardiogram 5 weeks earlier. The patient progressed to sustained ventricular tachycardia and persistent cardiogenic shock during placement of the valve prosthesis, followed by unsuccessful cardiopulmonary resuscitation.

**Table 3:** Procedural characteristics and COVID‐19 related outcomes

Procedural characteristics	Study population, *N* = 71
Valve type	
Abbott portico, *n* (%)	18 (25)
Medtronic Evolut R, *n* (%)	51 (72)
Edwards Sapien III, *n* (%)	2 (3)
Approach	
Femoral, n (%)	59 (83)
Subclavian, *n* (%)	10 (14)
Transapical, *n* (%)	2 (3)
Anesthesia	
General anesthesia, *n* (%)	29 (41)
Conscious sedation, *n* (%)	42 (59)
**Procedural outcomes**	
Total duration of hospitalization, days	5 (4–7)
Duration of hospitalization in TAVR center, days	2 (2–4)
Duration of hospitalization in referring hospital, days	3 (2–4)
Admission to ICU, *n* (%)	21 (30)
PVR ≥moderate, *n* (%)	7 (10)
Vascular complication, n (%)	8 (11)
Major, *n* (%)	2 (3)
Minor, *n* (%)	6 (8)
Bleeding complication, *n* (%)	8 (11)
Major, *n* (%)	2 (3)
Minor, *n* (%)	6 (8)
Stroke/TIA, *n* (%)	5 (7)
Conduction disturbance requiring pacemaker implantation, *n* (%)	6 (8)
Conversion to open surgery, *n* (%)	1 (1)
30‐Day mortality, *n* (%)	4 (6)
**COVID‐19 related outcomes**	
COVID‐19 testing before TAVR, n (%)	25 (35)
Positive testing (PCR and/or CT), n (%)	0
COVID‐19 testing after TAVR, n (%)	8 (11)
Positive testing (PCR and/or CT), n (%)	2 (3)
Death due to COVID‐19, n (%)	2 (3)

*Note*: Data are presented as median with interquartile range, or as number (%).

Abbreviations: COVID‐19, coronavirus disease 2019; CT, computed tomography; ICU, intensive care unit; PCR, polymerase chain reaction; PVR, paravalvular regurgitation; TAVR,  transcatheter aortic valve replacement; TIA, transient ischemic attack.

Of the remaining 70 patients, 21 involved admission to the ICU, mainly for postanesthesia recovery. Of these 21 patients, 18 were discharged to the CCU on the same day.

Patients were discharged to their referring center after a median stay of two days in our institution. In their referring center, median hospital stay was 3 days, resulting in a median total hospital stay of 5 days (IQR: 4–7 days). Besides the aforementioned case of procedural death, there was one patient that died 4 days after TAVR, which was complicated by device migration, for which a second valve prosthesis was placed. Moreover, the procedure was complicated by a cerebrovascular accident and a local dissection of the distal ascending aorta, for which conservative treatment was chosen. Four days after the TAVR procedure, the patient died after unsuccessful resuscitation for asystolic cardiac arrest. Autopsy and postmortem computed tomography (CT) showed a hemopericardium secondary to a rupture of the ascending aorta. In addition to the two beforementioned cases of intra‐ and postprocedural mortality, two patients died because of respiratory failure due to PCR proven COVID‐19 pneumonia. In conclusion, a total of 4 patients (6%) died within 30 days after TAVR. The two cases of respiratory failure due to COVID‐19 pneumonia after TAVR will be described in more detail below.

### 3.1 Case descriptions of two patients with COVID‐19 pneumonia after TAVR (Central Figure)

The first patient was a 76‐year‐old man referred to our hospital as an outpatient with critical AS (aortic valve area [AVA] of 0.5 cm^2^) and mild left ventricular dysfunction (LVEF: 40%–45%). His previous medical history was extensive, including chronic obstructive pulmonary disease (COPD), diabetes mellitus, multiple myocardial infarctions, and percutaneous coronary interventions, and quadruple coronary artery bypass grafting 8 years before. Due to his severe symptoms (graded as NYHA Class III–IV) with critical AS, this patient was triaged as urgency Level 1 (i.e., TAVR preferably within 1 week).

At admission, the patient did not exhibit any COVID‐19 related symptoms. Therefore, no COVID‐19 testing was performed beforehand, according to protocol. Using conscious sedation, TAVR was successfully performed using transfemoral approach, with trivial valvular and paravalvular regurgitation. Patient was discharged the following day to the referring hospital for further rehabilitation, and discharged home 3 days after TAVR.

Thirteen days after the TAVR procedure (and 10 days after last hospital stay), the patient was presented in our emergency department with a 4‐day history of fever, fatigue and dyspnea. Chest CT showed bilateral ground‐glass opacities and crazy paving appearance, typical of COVID‐19 pneumonia.^9^ Subsequent PCR‐testing was positive for COVID‐19. The patient was admitted and treated with oxygen and chloroquine. Rapid deterioration to hypoxemic respiratory failure warranted the initiation of invasive mechanical ventilation. Intubation and ICU admission was declined by both patient and family. The patient died the next morning. Regarding contact tracing, the patient received only hospital and house visits from two visitors. Visitor 1 exhibited loss of taste and smell (which later progressed to symptoms of dyspnea) 3 days before the patient displayed any COVID‐19 related symptoms (Central Figure). Visitor 2 exhibited symptoms of dyspnea 7 days after the patient's death (12 days after the patient first exhibited COVID‐19 related symptoms). In both the TAVR‐center and the referring hospital, no COVID‐19 cases were reported among the hospital personnel during that period.

The second patient was an 85‐year‐old man who was referred to our hospital as an outpatient with symptomatic, severe aortic stenosis (AVA 0.8 cm^2^) and preserved LVEF of more than 50%. His medical history included permanent atrial fibrillation and bilateral hip replacement. His symptoms were graded as NYHA II, with dyspnea on exertion and fatigue being his main symptoms. This patient was triaged as urgency Level 2.

Using conscious sedation, TAVR was successfully performed through transfemoral approach, with no valvular and paravalvular regurgitation. However, the procedure was complicated by bleeding of the right femoral artery due to unsuccessful placement of the closure device, necessitating surgical repair under general anesthesia and admission to the ICU. The same evening of the TAVR procedure, the patient was transferred to the CCU. The patient was discharged to the cardiology ward of the referring hospital for further rehabilitation the following day, and discharged home 4 days after TAVR.

Seven days after discharge (and 11 days after TAVR), the patient presented to the emergency department with a 2‐day history of fever, dyspnea and orthopnea. Chest CT showed bilateral ground‐glass opacities and possible peribronchial infection in the left lower lobe. Subsequent PCR testing was positive for COVID‐19. Patient was admitted to the COVID‐19 ward and died 5 days later due to respiratory failure.

Contact tracing revealed hospital visits from only one visitor for this patient. This person exhibited COVID‐19 related symptoms after the patient's readmission to the hospital (4 days after the patient exhibited symptoms), with PCR confirming COVID‐19 five days after the patient's death. Other visitors outside the hospital never exhibited any COVID‐19 related symptoms. In both the TAVR‐center and the referring hospital, no COVID‐19 cases were reported among the hospital personnel during that period.

## 4 DISCUSSION

In our single‐institutional cohort study with 71 patients undergoing TAVR during the COVID‐19 crisis, there were two patients (3%) with proven COVID‐19 pneumonia a few days after TAVR, both resulting in death approximately 2 weeks after TAVR (and 11–12 days after last hospital stay).

**Figure 1:**
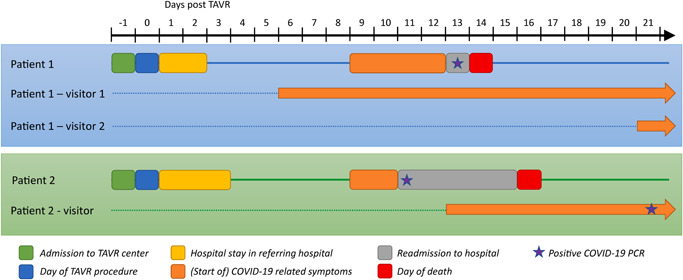
Central Figure : timeline of patients with COVID‐19 pneumonia after TAVR (accompanied by data on contact tracing). COVID‐19, coronavirus disease 2019; PCR, polymerase chain reaction; TAVR, transcatheter aortic valve replacement

Although it is clear that TAVR cannot be postponed for a prolonged period in patients with symptomatic severe or critical AS, the risk of deferment of the procedure has to be balanced against the dangers of COVID‐19 transmission before and after the procedure and associated morbidity and mortality in this vulnerable population. For inpatients who cannot be discharged due to medical reasons, we believe it is rational to perform the necessary procedures during the COVID‐19 crisis, as recommended by the previously mentioned ACC/SCAI consensus statement.^5^ However, for outpatients, there will be need for additional hospital admissions and hospital stay, potentially increasing their risks of COVID‐19 exposure. It has been well documented that the case‐fatality rate is significantly higher in elderly patients with COVID‐19 pneumonia (8%–13% for age 70–79 years, 15%–20% for age ≥80 years).^10^ Furthermore, the TAVR population is typically frail with important comorbid diseases. In our study population, 100% had coronary or peripheral artery disease, 37% had diabetes mellitus, 20% had cerebrovascular disorders, 21% had COPD, all which are known risk factors for increased COVID‐19 related morbidity and mortality.^11^, ^12^ In addition, TAVR might lead to significant inflammatory modulation,^13^, ^14^ as with most surgical and interventional procedures,^15^ leading to an exacerbated course of a COVID‐19 infection (Figure 1).

At this moment, the risks of nosocomial COVID‐19 exposure remain unknown and are currently under investigation (NCT04290780). Nevertheless, cases of nosocomial COVID‐19 transmission are factual and have already been reported.^16^, ^17^ In our study, two patients with COVID‐19 pneumonia died respectively 14 and 16 days after TAVR. Initial COVID‐19 symptoms started 5–6 days after discharge to home, and 9 days after TAVR procedure. Considering the reported median incubation period of 5 days between patients showing symptoms and initial COVID‐19 exposure,^18^ the patients can either have acquired COVID‐19 during hospital stay or at home. The exact source of COVID‐19 transmission remains uncertain. In our own center and the referring hospitals where the two patients were admitted, no COVID‐19 cases have been reported among the hospital personnel during that time period. Conversely, among the visitors of these two patients, one exhibited anosmia and ageusia before the patient demonstrated COVID‐19 related symptoms (Central Figure). Although visiting restrictions were already widely implemented in all hospitals in the Netherlands and worldwide, anosmia and ageusia were possible less well recognized COVID‐19 related symptoms in the general public at that time. Nevertheless, it might be advisable to encourage even stricter visiting guidelines for the patients who are at high risk for severe illness from COVID‐19, including these elderly patients with multiple comorbid diseases undergoing TAVR.

There are several limitations to our current study. First of all, only a minority of patients underwent COVID‐19 testing by PCR before TAVR (35%). Therefore, there is a possibility that the two COVID‐19 cases in our series were already COVID‐19 positive before their hospital admission and before TAVR. However, considering the time interval between hospital admission and onset of symptoms (10 days in both patients), it is not likely these patients would have tested positive before TAVR. Also, only eight patients underwent PCR testing after TAVR, thereby possibly underestimating the number of postprocedural COVID‐19 cases by neglecting the asymptomatic patients.

Second, the study population is too small to justify definitive conclusions regarding risks and route of COVID‐19 transmission and the additional morbidity and mortality risks caused by COVID‐19 infection. Nevertheless, to the best of our knowledge, this is the first study reporting data on feasibility and safety outcomes of TAVR procedures during the COVID‐19 pandemic. Data from large, preferably multicentered or nationwide registries are needed to better clarify these risks.

What are the implications of our findings? First of all, a continued TAVR program during this pandemic is feasible, despite restricted hospital resources and minimal ICU capacity. With a strategy focused on TAVR through conscious sedation when feasible, ICU dependency can be diminished. In our study, 61% of patients were assessed beforehand as feasible to undergo TAVR using conscious sedation, with one patient requiring conversion to general anesthesia due to a vascular complication necessitating surgical repair. This rate is similar to the recently reported proportion of 64% undergoing transfemoral TAVR under conscious sedation among 120,080 patients in the TVT Registry.^19^ Conversely, while a continued TAVR program is feasible on an organizational level, we want to highlight that the continuation of non‐COVID‐19 related care during this unprecedented pandemic in modern times is not without risks for the patient. This is especially true regarding TAVR for a particularly vulnerable, elderly population. During the study period, there was still active COVID‐19 transmission in the community. Through a combination of containment and mitigation activities, the number of new COVID‐19 cases are stabilizing or on the decline worldwide.^20^ The overall prevalence and transmission risks of COVID‐19 in the general population may now be much lower. Consequently, focus has already been shifting towards “post‐COVID‐19” reactivation of surgical and interventional programs.^21^ However, concern has also been raised regarding potential resurgence and possible additional COVID‐19 waves.^22^ Therefore, depending on the actual regional COVID‐19 prevalence and hospitalization rates, COVID‐19 related concerns will remain. As such, COVID‐19 associated risks of in‐hospital treatments with accompanied COVID‐19 transmission dangers should be an important focus of future reports, and these risks should be balanced against the hazards of deferred interventions for the cardiovascular patient on an individual basis. We expect this will be of continued concern for the foreseeable future.

## 5 CONCLUSION

In conclusion, we have reported the first case series of TAVR procedures performed during the COVID‐19 pandemic. Two cases of COVID‐19 pneumonia were diagnosed, with an unknown source of transmission soon after the intervention, both leading to mortality within 2 weeks after hospital discharge. We eagerly await subsequent reports from large registries to more accurately clarify the risks and source of COVID‐19 transmission after cardiac interventions, as well as the accompanied additional risks caused by COVID‐19 related morbidity and mortality. Until then, the complex balancing act of weighing the risks of health loss due to COVID‐19 against the risks of postponing a potentially life‐saving procedure remains a challenge for the clinician and the patient, and should be part of shared‐decision making. Furthermore, strict visiting policies should be considered in this vulnerable population after TAVR, both during and after hospital stay, with education of potential visitors of all COVID‐19 related symptoms.

## CONFLICT OF INTERESTS

The authors declare that there are no conflict of interests.
